# On Micro-Macro Transition in Non-Linear Mechanics

**DOI:** 10.3390/ma3010296

**Published:** 2010-01-08

**Authors:** Claude Stolz

**Affiliations:** Lab. Mécanique des Solides, Ecole polytechnique UMR 7649, 91128 Palaiseau Cedex France; E-Mail: stolz@lms.polytechnique.fr; Tel.: +33(0)1 69 33 58 05

**Keywords:** homogeneization, damage, elastoplasticity, concentration tensor

## Abstract

This paper is devoted to the description of the general relationships between microscopic and macroscopic mechanical quantities in non-linear mechanics. From a thermodynamical viewpoint, it is only necessary to know the two macroscopic potentials (macroscopic free energy and macroscopic potential of dissipation) to describe the state of the body and its quasistatic evolution. These global potentials are the averages of the local ones. We point out some particular cases of non-linearities, especially the case of damaged materials.

## 1. Introduction

This paper is devoted to the description of the general relationships between microscopic and macroscopic mechanical quantities in non-linear mechanics. Many studies have been dedicated to the relations between mechanical average quantities as stresses or strains in small or finite transformation ([1,2,3,4,5]).

Our purpose is to reformulate these relations in the framework of a thermodynamical point of view as proposed in [6]. This paper proposes some extensions of classical relations to nonlinear mechanics in small perturbations.

This thermodynamical point of view is useful to separate reversibility and irreversibility of the global response. The overall behaviour of a body, whose local properties are known, is the solution of a complicated boundary value problem, whose boundary conditions are specific.

Consider a small volume element of an heterogeneous material, two scales are distinguished on this volume. The microscopic one, where the properties vary from point to point as in a highly heterogeneous body, and the macroscopic one, where the properties are those of a homogeneous continuum.

In order to determine the overall behaviour with accuracy, it is essential to define the so-called representative volume element (RVE), which must be small enough to allow us to distinguish the microscopic heterogeneities and sufficiently large to be representative of the overall behavior. The scale of the RVE is chosen with respect to the scale of the heterogeneities and their interactions. A discussion can be found in [7] to specify the condition of the existence of such a RVE.

The local behaviour is determined by two thermodynamical potentials : the local free energy *w* is related to the equilibrium state and to the reversibility, and the potential of dissipation *d* which governs the irreversible processes.

To characterize the overall behaviour in a thermodynamical sense, it is only necessary to know the corresponding two macroscopic potentials: the macroscopic free energy *W* and the macroscopic potential of dissipation *D*.

The macroscopic free energy *W* is related with the equilibrium state and to the reversible part of the evolution, the potential of dissipation characterizes the irreversibility.

For sake of simplicity, we consider only isothermal processes or we assume in a more general case that the variation of temperature *τ* is uniform on the RVE. This condition is a necessary condition to determine the global free energy *W* of the body. This quantity is defined only for a thermodynamical state of equilibrium, which is a mechanical equilibrium state under uniform temperature. The two potentials are determined and simultaneously the quasistatic evolution of the system is analysed.

We propose to establish the relations between microscopic and macroscopic potentials. We characterize some macroscopic state variables, for example we define the decomposition of the macroscopic strain in reversible and irreversible parts.

Denoting the volume of the RVE by Ω, with any microscopic quantity *f*, we can associate its macroscopic value *F* by an averaging process on the RVE,

(1) $$F=\frac{1}{V}\int_{\Omega }fd\omega =<f>$$

By this way a unique macrostate quantity is defined for each microstate. However the macroscopic free energy at a given state is the total free energy at an equilibrium state. This state is the solution of a boundary value problem, with particular boundary conditions. To be efficient, these conditions must satisfy some properties, summarized in the concept of concentration process or localization process ([6,10]). The concentration process takes the bonding conditions between phases into account. The interface between phases is assumed to be perfect.

Successively, the modes of localization in small perturbation and applications are analyzed first in linear thermoelasticity, then in plasticity and in partially damaged materials as defined in [10].

## 2. Mode and Process of Localization

The mode of localization is defined by suitable boundary conditions and properties for the characterization of the bonding between phases.

We denote by $\underline{n}$ the unit normal to the boundary ∂Ω of Ω and we assume that $\partial \Omega =\partial \Omega_{T}\cup \partial \Omega_{u}$ where $\partial \Omega_{T}$ and $\partial \Omega_{u}$ are disjoint parts of ∂Ω, on which respectively the stress vector and the displacement vector are prescribed. The boundary conditions over ∂Ω must be chosen such that all equations of continuum mechanics are satisfied in a compatible manner with the averaging process.

### 2.1. Statical admissible stress field

The local stresses *σ* satisfy:

- the equations of equilibrium
  (2) divσ=0,overΩ
- the boundary conditions
  (3) $$\sigma .\underline{n}={\underline{T}}^{d}\mathrm{on}\partial \Omega_{T}$$

In the heterogeneous media the interface between phases is perfect, so that the stress vector is continuous along each interface Γ:

(4) $${\left[\sigma \right]}_{\Gamma }.\underline{\nu }=0,\mathrm{along}\Gamma$$

All stress fields *σ* satisfying these conditions (2,3,4) is called statical admissible (S.A.) with Σ=<σ> in the mode of localization if the boundary conditions is compatible with the averaging process

(5) $$\Sigma =<\sigma >=\frac{1}{V}\int_{\partial \Omega }{\left\{\sigma .\underline{n}⊗x\right\}}_{s}dS$$

### 2.2. Kinematical admissible fields

The local displacement $\underline{u}$ satisfies the boundary conditions $\underline{u}={\underline{U}}^{d}$ over $\partial \Omega_{u}$. The strain *ε* associated with this displacement is defined as

(6) $$\varepsilon =\frac{1}{2}\left(\nabla \underline{u}+\nabla^{T}\underline{u}\right),\varepsilon_{ij}=\frac{1}{2}\left(\frac{\partial {\underline{u}}_{i}}{\partial x_{j}}+\frac{\partial {\underline{u}}_{j}}{\partial x_{i}}\right)$$

over Ω, and the macroscopic strain is then deduced by

(7) $$E=<\varepsilon >=\frac{1}{V}\int_{\partial \Omega }\frac{1}{2}\left(\underline{u}⊗\underline{n}+\underline{n}⊗\underline{u}\right)dS$$

The displacement is continuous along all interfaces between phases

(8) $${\left[\underline{u}\right]}_{\Gamma }=0,\;\text{along}\;\Gamma$$

All strain fields *ε* satisfying these conditions will be said to be kinematically admissible (K.A.) with *E* in the mode of localization.

### 2.3. Hill-Mandel conditions of macrohomogeneity

The boundary conditions (${\underline{T}}^{d},{\underline{U}}^{d}$) must satisfy the hypothesis of macrohomogeneity in the sense of Hill-Mandel:

for any stress field $\sigma^{*}$ S.A. with $\Sigma^{*}=<\sigma^{*}>$ in the mode and any field $\varepsilon^{\prime }$ K.A. with $E^{\prime }=<\varepsilon^{\prime }>$ in the mode, we must have :

(9) $$\Sigma^{*}\;:E^{\prime }=<\sigma^{*}\;:\varepsilon^{\prime }>$$

### 2.4. The process of localization

By adding the knowledge of the local constitutive law, we can study the evolution of the system for a given history of the prescribed boundary conditions. But the determination of a macroscopic behaviour requires that the process of localization, defined by a mode of localization and local constitutive law, ensures existence and uniqueness of the microscopic fields. In such a case we can deduce the form of the macroscopic constitutive law in the following way. For given macroscopic quantities we solve the boundary value problem associated with the process of localization and then the local fields are determined. Finally using the averaging process we find the unknown macroscopic quantities.

### 2.5. Particular mode of localization

There exist three particular well-known modes of localization for which the boundary conditions, the average process and the Hill-Mandel conditions are simultaneously verified.

The first one is the concentration process under macrohomogeneous stresses ${\underline{T}}^{d}=\Sigma .\underline{n}$ over ∂Ω, where Σ is a second order symetric tensor. Then for all $\sigma^{*}$ S.A. in the mode, $\Sigma^{*}$ must be equal to Σ. The displacement ${\underline{u}}^{\prime }$ is closed to ${\underline{U}}^{\prime }=E^{\prime }.x$ over ∂Ω

(10) $$\int_{\partial \Omega }\left({\underline{u}}^{\prime }-{\underline{U}}^{\prime }\right)⊗\underline{n}dS=0$$

This is obtained by taking $\sigma^{*}=\Sigma$ in the Hill-Mandel condition, and then we have $E^{\prime }=<\varepsilon \left({\underline{u}}^{\prime }\right)>$.

Secondly, a concentration process under macrohomogeneous strain ${\underline{U}}^{d}=E.x$ over ∂Ω can be chosen. All kinematical fields ${\underline{u}}^{\prime }$ verify automatically the average condition on strains $E=<\varepsilon ({\underline{u}}^{\prime })>$ , and for any $\sigma^{*}$ statically admissible field we obtain from the macrohomogenous condition the average condition on stresses $\Sigma^{*}=<\sigma^{*}>$.

The third mode is the periodic description. The RVE is reduced to the geometry of an elementary cell. Choosing ${\underline{T}}^{d}$ as an antiperiodic function over $\partial \Omega_{T}$, and ${\underline{U}}^{d}$ a periodic field over $\partial \Omega_{u},$ the average condition on stresses is due to the equilibrium, the average condition on strains is ensured by the compatibility of the local strain, and the Hill-Mandel macrohomogeneous condition is deduced from the periodicity ([11,12]).

## 3. Potentials and General Properties

The local behavior is defined by the local free energy w(ε,α,τ), where *ε* is the strain, *α* represents a set of internal variables and *τ* is the variation of temperature. The state equations are given by

(11) $$\sigma_{R}=\frac{\partial w}{\partial \varepsilon },\;A=-\frac{\partial w}{\partial \alpha },s=-\frac{\partial w}{\partial \tau }$$

$\sigma_{R}$ is the reversible stress, *A* is the thermodynamical force associated with the evolution of *α* and *s* is the entropy. The Clausius-Duhem inequality of entropy production is reduced to

(12) $$D=\sigma :\dot{\varepsilon }-\left(\dot{w}+s\dot{\tau }\right)\geq 0$$

where the stresses *σ* are in equilibrium inside the body. Then

(13) $$D=\left(\sigma -\sigma_{R}\right):\dot{\varepsilon }+A\;\dot{\alpha }\geq 0$$

Two sources of dissipation appear, one is due to viscosity with the thermodynamical force $\sigma_{ir}=\sigma -\sigma_{R}$, the other one is associated with the evolution of internal variables.

To solve the problem of evolution, a complementary law is needed. The irreversible processes are driven by a potential of dissipation $d(\dot{\varepsilon },\dot{\alpha })$, which is a convex function of its arguments, the thermodynamical forces ($\sigma_{ir},A$) satisfy the normality rule :

(14) $$\left(\sigma_{ir},A\right)\in \partial d\left(\dot{\varepsilon },\dot{\alpha }\right)$$

this corresponds to the property:

(15) $$\forall \left({\dot{\varepsilon }}^{*},{\dot{\alpha }}^{*}\right),d\left(\dot{\varepsilon },\dot{\alpha }\right)+\sigma_{ir}:\left(\varepsilon^{*}-\dot{\varepsilon }\right)+A\;\left({\dot{\alpha }}^{*}-\dot{\alpha }\right)\leq d\left({\dot{\varepsilon }}^{*},{\dot{\alpha }}^{*}\right)$$

We assume henceforth that the local behaviour has no viscosity, $\sigma_{ir}$ vanishes then the reversible stress satisfies the conservation of momentum.

### 3.1. The boundary value problem of localization

We prescribe a macroscopic strain *E* and a uniform variation of temperature *τ* for a given distribution of internal parameters *α* over the RVE.

A solution of the boundary value problem in terms of displacement $\underline{u}$ or stresses *σ* is given by functions of *E* and *α* satisfying the set of equations:

- the local stresses are statical admissible
  (16) $$\mathrm{div}\sigma =0,\sigma .\underline{n}={\underline{T}}^{d}\mathrm{over}\partial \Omega_{T}$$
- the strain *ε* is kinematical admissible in the mode :
  (17) $$E=<\varepsilon \left(\underline{u}\right)>,\;\underline{u}={\underline{U}}^{d}\;\text{over}\;\partial \Omega_{u}$$
- the interface Γ is perfect, then the stress vector and the displacement are continuous:
  (18) $${\left[\sigma \right]}_{\Gamma }.\underline{\nu }=0,{\left[\underline{u}\right]}_{\Gamma }=0$$
- the stress and the strain are linked by the constitutive law
  (19) $$\sigma =\frac{\partial w}{\partial \varepsilon }\left(\varepsilon \left(\underline{u}\right),\alpha ,\tau \right)$$

If the local free energy *w* is a convex function of *ε* when *α* and *τ* are prescribed, the solution $\underline{u}$ of this boundary value problem is unique.

### 3.2. The global free energy

The macroscopic free energy *W* is defined by the value of the average

(20) $$W\left(E,\alpha ,\tau \right)=<w(\varepsilon \left(\underline{u}\right),\alpha ,\tau )>$$

where $\underline{u}$ is the solution of the boundary value problem of localization, *α* and *τ* being given at the equilibrium state. Then, from the Hill-Mandel macrohomogeneity condition we deduce the macroequation of state:

(21) $$\frac{\partial W}{\partial E}=<\frac{\partial w}{\partial \varepsilon }:\frac{\partial \varepsilon }{\partial E}>=<\sigma :\left(I+\frac{\partial \eta }{\partial E}\right)>=<\sigma >:<\left(I+\frac{\partial \eta }{\partial E}\right)>$$

Noting that *ε* is written as E+η, with *η* is a kinematical admissible strain such that <η>=0, then we have $\frac{\partial \eta }{\partial E}=0$ and hence the macroscopic stress *σ* is related to the macroscopic strain by the state equation

(22) $$\frac{\partial W}{\partial E}=<\sigma >=\Sigma$$

The macrostress at equilibrium is defined in the same way as the microstress, owing to the definition of the macroscopic thermodynamical potential *W*.

For a perturbation of temperature δτ, the variation of energy is

(23) $$-\frac{\partial W}{\partial \tau }\;\delta \tau =-<\frac{\partial w}{\partial \tau }\;\delta \tau >=<s>\delta \tau =S\;\delta \tau$$

then, the global entropy *S*, average of the local one, is related to the variation of the global free energy.

The other state equations are expressed as

(24) $$\underline{A}•\delta \underline{\alpha }=-\int_{\Omega }\frac{\partial w}{\partial \alpha }\;\;\delta \alpha \;d\Omega =-\frac{\partial W}{\partial \underline{\alpha }}•\delta \underline{\alpha }$$

The internal state in a global description for the system is defined by the value of α(x) at each point of Ω. At the macroscale the internal state is defined by a field of internal variables. This interpretation is emphasized by considering the potential of dissipation.

### 3.3. The global dissipation function

If the evolution of the internal parameters is given by a potential of dissipation $d(\dot{\alpha })$, convex function of $\dot{\alpha }$, the thermodynamical forces *A* are defined by the normality rule $A\in \partial d(\dot{\alpha })$. We define the global dissipation function as the function $D\left(\underline{\dot{\alpha }}\right)=<d\left(\dot{\alpha }\right)>$ of the field of internal parameters $\underline{\dot{\alpha }}$. The expression of the normality rule is transposed in terms of fields by integration over Ω :

(25) $$\forall {\dot{\alpha }}^{*},\;D\left(\underline{\dot{\alpha }}\right)+<A\left({\dot{\alpha }}^{*}-\dot{\alpha }\right)>\leq D\left({\underline{\dot{\alpha }}}^{*}\right)$$

It is obvious that *D* is a functional of $\dot{\alpha }$ and *A* is a linear form $<A\alpha^{*}>$ on fields $\alpha^{*}$ defined over Ω. Then the normality rule is written in terms of fields

(26) $$\underline{A}\in \partial D\left(\underline{\dot{\alpha }}\right)$$

In a general point of view, the governing equations for the macrostate have the same form as the governing equations for the microstate. The set of internal variables is replaced by a set of fields of internal variables.

For the overall behaviour, the value of internal state at each material point of Ω must be known.

### 3.4. Macrohomogeneous body and linear elasticity

For linear elasticity, the macroscopic elastic modulus has not the same value when macrohomogeneous strain or stress conditions are prescribed on the boundary ∂Ω. But when the body is macrohomogeneous in the sense of Hill-Mandel ([2,13,14]) the difference between the two moduli vanishes. More details could be found in [7] or in [15] about the relations between the definition of the RVE and the macrohomogeneity condition.

Assuming that all constituent phases are linear elastic, the local free energy density is given by $w\left(\varepsilon \right)=\frac{1}{2}\varepsilon :C\left(x\right):\varepsilon$, where C depends on the point *x* of Ω. The displacement $\underline{u}$ solution of the boundary value problem minimizes the potential energy of the system, the solution is unique. When one prescribes homogeneous strain condition ($\underline{u}=E.y$ for *y*∈∂Ω), the potential energy is reduced to *W*. The displacement $\underline{u}$ depends only on the given macroscopic value *E* and on the spatial distribution of the mechanical phases. The local stress *σ* is obtained as the solution of a problem of heterogeneous elasticity.

The boundary value problem for heterogeneous linear isotropic material is linear, the solution of this problem depends linearly on the boundary conditions. This proves the existence of concentration tensors A for stresses and B for strains. The Green functions L,M for the displacement $\underline{u}$ and the concentration tensors are such that

$$\begin{array}{ccc} \sigma & = & A:\Sigma ,\;\varepsilon =B:E\\ \underline{u} & = & L:E,\;B=\frac{1}{2}\left(\nabla L+\nabla^{T}L\right)\\ \underline{u} & = & M:\Sigma \end{array}$$

The tensor A is statical admissible with *I* in the mode of localization, dually the concentration tensor B is kinematical admissible with *I* in the mode of localization.

For macrohomogenous body, we can also define the effective modulus of elasticity Σ=<σ>=C:E. Then

<σ>=<C:ε>=<C:B>:E

then

(27) C=<C:B>

### 3.5. Properties of the concentration tensors

For fixed subscripts (p,q), $A_{ijpq}$ satisfies the equilibrium equations and homogeneous boundary conditions

$$\begin{array}{ccc} A_{ijpq,j} & = & 0,\text{on}\;\Omega \\ A_{ijpq}n_{j} & = & \frac{1}{2}\left(n_{p}\delta_{iq}+n_{q}\delta_{ip}\right)\mathrm{over}\partial \Omega \end{array}$$

The strain $\varepsilon_{E}=S:A:\Sigma =B:E$ satisfies the condition of compatibility ($S=C^{-1}$) and the relations between micro and macro scales can be defined

$$\begin{array}{ccc} \Sigma & = & C:E,\;C=<B^{T}:C:B>\\ S & = & <A^{T}:S:A>=C^{-1} \end{array}$$

We have used the notation ${\left(A^{T}\right)}_{ijpq}=A_{pqij}$. Moreover, we have the set of relations :

$$\begin{array}{ccc} S:A & = & B:S,\;A:C=C:B\\ <A> & = & I,<B>=I \end{array}$$

### 3.6. Estimation and bounds of elastic moduli

For composite materials estimation of strain or stress averages on each material phase can be obtained by solving specific problem of inclusions embedded an homogeneous body of characteristics $C_{o}$.

(28) $$C_{o}=3\kappa_{o}K+2\mu_{o}J,\;K_{ijpq}=\frac{1}{3}\delta_{ij}\delta_{pq},J=I-K$$

Using theorem of minimum of potential energy Hashin-Shtrickman bounds provide rigorous upper ($HS^{+}$) and lower bounds ($HS^{-}$) for the effective properties of composites. Initially developed for isotropic phases with overall isotropy [16], they were extended for some anisotropic media with ellipsoidal symmetry as defined in [17].

When the individual phases are isotropic and when the composite has overall isotropy, the spherical inclusion Eshelby’s solution is used to obtained explicit bounds. The averaged strain inside the inclusion of material *i* is given by

$$\varepsilon_{i}={\left(I+E_{o}:\left(C_{i}-C_{o}\right)\right)}^{-1}:\varepsilon_{\infty }$$

where $\varepsilon_{\infty }$ is deduced from the relation $E=\sum_{i}f_{i}\varepsilon_{i}$ with $f_{i}$ the volume fraction of material *i*.

$$E_{o}=\frac{\alpha_{o}}{3\kappa_{o}}K+\frac{\beta_{o}}{2\mu_{o}}J$$

with

$$\;\beta_{o}=\frac{6(\kappa_{o}+2\mu_{o})}{5(3\kappa_{o}+4\mu_{o})};\;\alpha_{o}=\frac{3\kappa_{o}}{3\kappa_{o}+4\mu_{o}}$$

Then the effective moduli are estimated by

$$\begin{array}{ccc} \kappa^{est}\left(\kappa_{o},\mu_{o}\right) & = & \frac{<\kappa {\left(1+\alpha_{o}\frac{\left(\kappa -\kappa_{o}\right)}{\kappa_{o}}\right)}^{-1}>}{<{\left(1+\alpha_{o}\frac{\left(\kappa -\kappa_{o}\right)}{\kappa_{o}}\right)}^{-1}>}\\ \mu^{est}\left(\kappa_{o},\mu_{o}\right) & = & \frac{<\mu {\left(1+\beta_{o}\frac{\left(\mu -\mu_{o}\right)}{\mu_{o}}\right)}^{-1}>}{<{\left(1+\beta_{o}\frac{\left(\mu -\mu_{o}\right)}{\mu_{o}}\right)}^{-1}>} \end{array}$$

where $<q>=\sum_{i}f_{i}q_{i}$ with $f_{i}$ the volume fraction of phase *i*.

The bulk modulus and shear modulus for any isotropic material are bounded by 0 and *∞*. For these values of $\kappa_{o},\mu_{o}$ the estimations $\kappa^{est},\mu^{est}$ are bounded by the classical bounds of Voigt and Reus as the effective values.

$$<\frac{1}{\mu }>\leq \mu^{eff}\leq <\mu >,<\frac{1}{\kappa }>\leq \kappa^{eff}\leq <\kappa >$$

This suggest to optimize for specific composite the choice of $C_{o}$ to have an estimation closed to the effective value. For example, under the hypotheses of overall isotropy and isotropic distribution of phases, the Hashin-Shtrikman bounds are obtained. The $HS^{+}$ upper bound is obtained for $\mu_{o}=\max_{i}\mu_{i},\;\kappa_{o}=max_{i}\kappa_{i}$ and similarly the $HS^{-}$ lower bounds with $\mu_{o}=\min_{i}\mu_{i},\;\kappa_{o}=min_{i}\kappa_{i}$.

- For a two-phase composite with $\left(\kappa_{2}<\kappa_{1},\mu_{2}<\mu_{1}\right)$ we have the estimation
  $$\kappa^{est}\left(\kappa_{o},\mu_{o}\right)=\frac{3\kappa_{1}\kappa_{2}+4\mu_{o}<\kappa >}{4\mu_{o}+3\kappa_{1}\kappa_{2}<\frac{1}{\kappa }>},\;\mu^{est}\left(\kappa_{o},\mu_{o}\right)=\frac{\left(9\mu_{o}\kappa_{o}+8\mu_{o}^{2}\right)<\mu >+6\mu_{1}\mu_{2}\left(\kappa_{o}+2\mu_{o}\right)}{9\kappa_{o}\mu_{o}+8\mu_{o}^{2}+6\mu_{1}\mu_{2}\left(\kappa_{o}+2\mu_{o}\right)<\frac{1}{\mu }>}$$
  then the HS bounds are
  $$\mu^{HS^{+}}=\mu^{est}\left(\kappa_{1},\mu_{1}\right),\mu^{HS^{-}}=\mu^{est}\left(\kappa_{2},\mu_{2}\right);\;\kappa^{HS^{+}}=\kappa^{est}\left(\kappa_{1},\mu_{1}\right),\kappa^{HS^{-}}=\kappa^{est}\left(\kappa_{2},\mu_{2}\right)$$
- When the composite microstructure is an assemblage of composite spheres bounds are closer than classical Hashin-Shtrikman bounds and improved in a general way using morphological patterns approach [18]. In the case of two incompressible phases, the lower bound for isotropic two-phases composites assemblages is obtained as previously and the upper bound is given by
  (29) $$\mu^{HSZ}=\mu_{2}(1+f_{1}F\left(f_{1},\gamma ,\gamma \right);\;\gamma =\frac{\mu_{1}}{\mu_{2}}$$
  where *F* is given by
  $$\frac{1}{F(c,\gamma ,\gamma_{o})}=\frac{2}{5}\left(1-c\right)+\frac{1}{1-\gamma }-\frac{c{\left(1-c^{2/3}\right)}^{2}}{\frac{10}{21}\frac{(}{1}9\left(1-\gamma \right)}16+19\gamma c^{7/3}+\frac{10}{21}+\frac{25}{24(\gamma_{o}-1)}$$
- For any isotropic phases, the general solution is given in [18], generalized description is also presented in [19] and bounds HSZ are derived for composite sphere assemblage (CSA) of Hashin [20].

### 3.7. More complex behaviour

For a more complex behaviour, we can solve the problem of localization with fixed (α,τ) ; the solution associated with a variation of the macroscopic strain dE is then an elastic response. The solution of this problem of heterogeneous elasticity is written as

$$\begin{array}{ccc} d\varepsilon & = & B\;:dE\\ d\sigma & = & A\;:d\Sigma =C:d\varepsilon \end{array}$$

here the value C(x) is the local instantaneous modulus of elasticity $\frac{\partial {}^{2}w}{\partial \varepsilon \partial \varepsilon }$. Then the concentration tensors are associated with these reversible tangent moduli for which we can define a macroscopic tangent modulus satisfying the general relation

(30) $$C=<B^{T}\;:C\;:B>$$

## 4. On the Decomposition of the Macroscopic Strain

Let Σ be the real macrostress and *σ* the corresponding microscopic one. The local solution during purely elastic behavior is as previously $\sigma_{E}=A\;:\Sigma$. The stress field $r=\sigma -\sigma_{E}$ is then self equilibrated.

In small strain, the total deformation *ε* is the sum of the elastic strain $\varepsilon_{e}$ and some initial strain $\varepsilon_{i}$. The elastic strain is related to *σ* by the constitutive law ($\varepsilon_{e}=S\;:\sigma$). The initial strain $\varepsilon_{i}$ induces an internal stress field *r* such that the local strain $\varepsilon_{res}$ satisfies the compatibility conditions and the constitutive behaviour

(31) $$\varepsilon_{res}=S:r+\varepsilon_{i}$$

The macroscopic elastic strain $E_{E}$ is the strain recovered by a purely elastic unloading, which corresponds locally to the interpretation of $\sigma_{E}$. The local strains *ε* and $\varepsilon_{E}=S\;:\sigma_{E}$ are kinematically admissible respectively with *E* and $E_{E}$ in the mode of localization. From the Hill-Mandel condition applied with A:<σ>, which is statically admissible with <σ> in the mode of localization, we obtain :

(32) $$E_{E}=<A^{T}\;:\varepsilon_{E}>,\;E=<A^{T}\;:\varepsilon >$$

Then the definition of the macroscopic modulus is recovered as $S=<A^{T}\;:S\;:A>$. The difference $\varepsilon -\varepsilon_{E}$ is a kinematically admissible field associated with the anelastic part $E_{res}$ of the macroscopic strain ($E_{res}=E-E_{E}$), and we obtain

(33) $$E_{res}=<\varepsilon_{res}>=<A^{T}\;:\varepsilon_{res}>=<A^{T}\;:\varepsilon_{i}>$$

Since *r* is a self equilibrated stress field and S:A is a kinematically admissible field, then <r:S:A>=0, this property is used to established the second equality. The thermodynamical interpretation of $E_{res}$ must be investigated, it depends on the local meaning of the strain $\varepsilon_{i}$ and of its evolution.

## 5. Transformation along a Moving Surface

During loading history, damage in continuum mechanics can be induced by the initiation and the growth of microcavities and microcracks. The description of damaged is based on the evolution of the microscopic properties, taking the growth of such degradation into account, through the idea that when some threshold value of stress, strain or embedded energy is reached, the material can’not support further tensile loading.

A connection with fracture mechanics can be made in an asymptotic sense [21] or in a hierarchic description [22]. Variational formulation were performed to describe the evolution of the surface between the sound and the damaged materials ([23,24]). Some particular case of homogenization is proposed by Petryck [26] for this analysis. In the case of elastic brittle materials or partially damaged materials some previous results have been obtained ([10,25]).

At each time the domain Ω is composed of two distinct volumes $\Omega_{1}$ and $\Omega_{2}$ which are occupied by two materials with different mechanical characteristics. The interface between the two phases is perfect and denoted by Γ. The phase 1 changes into the phase 2 in an irreversible manner due to the mechanical loading along the moving surface Γ, defined by an equation of the form S(x,t)=0. The extension of the phase 2 is related to this moving surface, the equation of the surface is obtained in an explicit manner depending on the history of the loading. In order to study the general formulation of the relationships between microscopic mechanical fields and macroscopic quantities we do not discuss the characteristics of the evolution of the interface, and at each point of the interface we consider that the normal velocity *φ* is determined.

When the interface moves, the evolution of any macroscopic quantity *F* is given by:

(34) $$\dot{F}=<\dot{f}>-\frac{1}{\Omega }\int_{\Gamma }{\left[f\right]}_{\Gamma }\phi dS$$

where ${\left[f\right]}_{\Gamma }\left[f\right]=f_{1}-f_{2}$ is the jump of the quantity *f* at a point of Γ , $\underline{n}$ is the normal vector to Γ external to phase 1. As the interface is moving, the transport condition for any mechanical quantity *f* at a geometrical point of Γ is given by the convected derivative $D_{\phi }f$

(35) $$D_{\phi }f=\lim_{\begin{array}{c} \Delta t\to 0 \end{array}}\frac{f(x+\phi n\Delta t,t+\Delta t)-f(x,t)}{\Delta t}$$

Along Γ, the displacement and the stress vector are continuous, then their rates satisfy the compatibility equations of Hadamard

(36) $${\left[D_{\phi }\left(\sigma .\underline{n}\right)\right]}_{\Gamma }=0,\;D_{\phi }{\left[\underline{u}\right]}_{\Gamma }={\left[v\right]}_{\Gamma }+\phi {\left[\nabla \underline{u}\right]}_{\Gamma }.\underline{n}=0$$

So, we must take into account these discontinuities. As the displacement is continuous along Γ, ${\left[\underline{u}\right]}_{\Gamma }=0$, then the gradient along Γ of the displacement $\nabla_{\Gamma }\underline{u}$ is continuous. If $\underline{n}$ is the normal vector of the surface Γ, the discontinuity of the gradient of the displacement has the form

(37) $${\left[\nabla \underline{u}\right]}_{\Gamma }=\lambda ⊗\underline{n}$$

The stress vector is continuous, ${\left[\sigma \right]}_{\Gamma }.\underline{n}=0$. Combining all the property of continuities, the discontinuities of *σ* and $\nabla \underline{u}$ have a property of orthogonality:

(38) $${\left[\sigma \right]}_{\Gamma }:{\left[\nabla \underline{u}\right]}_{\Gamma }=0$$

### 5.1. Case of linear elasticity

In this section, the two phases are linear elastic media. At time t, the distribution of the phases is known and the localization process is defined by the equilibrium state of a heterogeneous elastic medium. The displacement $\underline{u}$, at equilibrium, satisfies the equations of the boundary value problem associated with the mode of localization. At each time the tensors of concentration are defined and the macroscopic behaviour is obtained as previously by

(39) $$E=<A^{T}:S:A>:\Sigma =S:\Sigma$$

Between time t and t+dt, the concentration and the shape of the phases have changed, then the concentration tensors A and B have evolution associated with the normal velocity *φ* of propagation of the interface. The variation of the geometry of the phases induces a variation of the elastic moduli. For a macroscopic evolution of the loading, the phase l is transformed into the phase 2 along some parts of Γ. The rate of A, denoted by $\dot{A}$, is linked to the normal velocity of propagation, the same is true for $\dot{M}$. So the local response is

(40) $$\dot{\sigma }=A:\dot{\Sigma }+\dot{A}:\Sigma ,\;v=M:\dot{\Sigma }+\dot{M}:\Sigma$$

The rates of the concentration tensors verify Hadamard’s relations on Γ:

(41) $${\left[D_{\phi }\left(A.\underline{n}\right)\right]}_{\Gamma }=0,{\left[D_{\phi }M\right]}_{\Gamma }=0$$

The global evolution of the macroscopical quantities are then deduced, using the hypothesis of macrohomogeneity:

(42) $$\dot{\Sigma }=<\dot{\sigma }>-\frac{1}{\Omega }\int_{\Gamma }{\left[\sigma \right]}_{\Gamma }]\;\phi dS$$

(43) $$\dot{E}=<A^{T}:\dot{\varepsilon }>-\frac{1}{\Omega }\int_{\Gamma }A^{T}:{\left[\nabla u\right]}_{\Gamma }\;\phi dS$$

In a similar way, the variation of the elastic moduli is given by

(44) $$\dot{S}=\frac{1}{\Omega }\int_{\Gamma }2g\;\phi \;dS,\;g=\frac{1}{2}{\left[A^{T}:S:A\right]}_{\Gamma }-A^{T}:{\left[\nabla M\right]}_{\Gamma }$$

where g is the density of energy release rate.

#### Total dissipation

The total energy $W=\frac{1}{2}\Sigma :S:\Sigma$ arises to the macroscopic dissipation

$$\begin{array}{ccc} D_{m} & = & \frac{1}{2}\Sigma :\dot{S}:\Sigma =\frac{1}{\Omega }\int_{\Gamma }G\;\phi dS\geq 0\\ G & = & \Sigma :g:\Sigma \end{array}$$

the quantity *G* is the energy release rate defined along Γ . So even if the local behavior is reversible, the propagation of a surface of discontinuity inside the body generates dissipation. The macroscopic behaviour has variable elastic moduli.

### 5.2. Internal stresses

More generally, when both materials are elastoplastic or with initial strains, because of the existence of incompatible strains, a self equilibrated stress field *r* appears, and the local stress can be decomposed as

(45) $$\sigma =A:\Sigma +r=\sigma_{E}+r$$

The field *r* being self-equilibrated the following relations are obtained

$$\begin{array}{ccc} & < & r>=0,{\left[r\right]}_{\Gamma }.\underline{n}=0\\ r.\underline{n} & = & 0\mathrm{along}\partial \Omega ,\;\mathrm{div}r=0\mathrm{over}\Omega \end{array}$$

The local strain *ε* is related to the displacement $\underline{u}$ K.A. with *E*. Let us denote by $\varepsilon_{e}$ the elastic strain

(46) $$\varepsilon_{e}=\varepsilon -\varepsilon_{p}=S:\sigma$$

Let us introduce two other displacement fields:

- the first one ${\underline{u}}_{E}=M:\Sigma$ is K.A with $E_{E}=S:\Sigma$ and defines the strain
  (47) $$\varepsilon_{E}=\varepsilon \left({\underline{u}}_{E}\right)=S:\sigma_{E}$$
- the second one ${\underline{u}}_{ir}$ is K.A. with $E_{ir}=<\varepsilon_{ir}>$ and defines the strain
  (48) $$\varepsilon_{ir}=\varepsilon \left({\underline{u}}_{ir}\right)=\varepsilon_{p}+S:r$$

Thanks to these definitions, one obtains:

$$\begin{array}{ccc} \varepsilon & = & \varepsilon_{e}+\varepsilon_{p}=S:A:\Sigma +S:r+\varepsilon_{p}=\varepsilon_{E}+\varepsilon_{ir}\\ \underline{u} & = & {\underline{u}}_{E}+{\underline{u}}_{ir}=M:\Sigma +{\underline{u}}_{ir}\\ E & = & E_{E}+E_{ir} \end{array}$$

For a given macroscopic evolution $\dot{\Sigma }$, the plastic strain rate can evolve and the propagation of Γ can occur. The evolution of the state obeys to the decomposition

(49) $$\dot{\varepsilon }={\dot{\varepsilon }}_{p}+S:\dot{\sigma },\;\dot{\sigma }={\dot{\sigma }}_{E}+\dot{r}$$

In the previous relations, ${\dot{\sigma }}_{E}$ corresponds to the microscopic variation for a purely elastic behaviour get with the same propagation of the interface Γ. So, it gives

(50) $${\dot{\sigma }}_{E}=A:\dot{\Sigma }+\dot{A}:\Sigma$$

where the localization tensor A verifies the Hadamard’s compatibility equations. The rate of each displacement have discontinuities according to the continuity compatibility equations

(51) $$D_{\phi }{\left[\underline{u}\right]}_{\Gamma }=0,D_{\phi }{\left[{\underline{u}}_{E}\right]}_{\Gamma }=0,D_{\phi }{\left[{\underline{u}}_{ir}\right]}_{\Gamma }=0$$

The application of the Hill-Mandel hypothesis to these displacement fields and to the related strain fields gives a set of relations

$$\begin{array}{ccc} \dot{E} & = & <A^{T}:\dot{\varepsilon }>-\frac{1}{\Omega }\int_{\Gamma }A^{T}:{\left[\nabla \underline{u}\right]}_{\Gamma }\;\phi dS\\ {\dot{E}}_{E} & = & <A^{T}:{\dot{\varepsilon }}_{E}>-\frac{1}{\Omega }\int_{\Gamma }A^{T}:{\left[\nabla {\underline{u}}_{E}\right]}_{\Gamma }\;\phi dS=S:\dot{\Sigma }+\dot{S}:\Sigma \end{array}$$

By substraction, it allows us to define the variation of the irreversible strain

(52) $${\dot{E}}_{ir}=<A^{T}:{\dot{\varepsilon }}_{ir}>-\frac{1}{\Omega }\int_{\Gamma }A^{T}:{\left[\nabla {\underline{u}}_{ir}\right]}_{\Gamma }\;\phi dS$$

or

(53) $${\dot{E}}_{ir}=<A^{T}:{\dot{\varepsilon }}_{p}>+<A^{T}:S:\dot{r}>-\frac{1}{\Omega }\int_{\Gamma }A^{T}:{\left[\nabla {\underline{u}}_{ir}\right]}_{\Gamma }\;\phi dS$$

Since the residual stress *r* is a self equilibrated field, we get from $<r>=0,{\left[r\right]}_{\Gamma }.\underline{n}=0$,

$$\begin{array}{ccc} 0 & = & <\dot{r}>-\frac{1}{\Omega }\int_{\Gamma }{\left[r\right]}_{\Gamma }\;\phi dS\\ 0 & = & {\left[D_{\phi }\left(r.\underline{n}\right)\right]}_{\Gamma } \end{array}$$

Hence, the rate of internal stresses $\dot{r}$ is not self-equilibrated and the macroscopic irreversible strain takes the form

(54) $${\dot{E}}_{ir}=<A^{T}:{\dot{\varepsilon }}_{p}>-\frac{1}{\Omega }\int_{\Gamma }A^{T}:{\left[\nabla {\underline{u}}_{ir}\right]}_{\Gamma }\;\phi dS+\frac{1}{\Omega }\int_{\Gamma }{\left[r\right]}_{\Gamma }:\nabla M\;\phi dS$$

The irreversible part of the macroscopic strain is decomposed into two parts: one due to the volume irreversibility, the other due to the variation of the residual stress field, which is essentially dependent with the geometry of the phases. Even if the internal strain has no evolution, there exists an irreversible macroscopic strain due to the variation of internal stresses essentially dependent on the evolution of the geometry of phases.

#### Dissipation

In the case of a plastic behaviour, the free energy of the system takes the form $W\left(\Sigma ,\varepsilon_{p},\alpha \right)=\frac{1}{2}<\sigma :\left(\varepsilon -\varepsilon_{p}\right)>+h\left(\alpha \right)$ where *α* is any internal variable.

The embedded energy associated with the residual stresses *r* is then $W_{b}=\frac{1}{2}<r:S:r>$ . Thus the dissipation rate is

(55) $$D_{m}=\Sigma :{\dot{E}}_{ir}-{\dot{W}}_{b}+\frac{1}{2}\Sigma :\dot{S}:\Sigma -\frac{\partial h}{\partial \alpha }\dot{\alpha }\geq 0$$

The expression of $D_{m}$ in term of local quantities is

(56) $$D_{m}=<\sigma :{\dot{\varepsilon }}_{p}>-\frac{\partial h}{\partial \alpha }\dot{\alpha }+\frac{1}{\Omega }\int_{\Gamma }\left({\left[w\right]}_{\Gamma }-\sigma :{\left[\nabla \underline{u}\right]}_{\Gamma }\right)\phi dS$$

In this form two parts are distinguished ; the first one is related to the plastic effects, the second one is related to the moving surface. The part of the dissipation due to plasticity and hardening in not directly related to the irreversible strain. These equations shows that the main difficulty in a macroscopic approach is to determine the relative part due to plasticity and to local rupture in macroscopic tests.

#### Case of plasticity

The case of plasticity is recovered when no transformation exist along Γ. In the dissipation, two kinds of hardening are then present: the hardening due to the incompatibility of the plastic strain, the self-hardening of each constituent. The hardening is described in the energy embedded in the residual stresses and in the self-hardening energy, which emphasizes the role of the embedded energy on the hardening.

#### Case of damaged material

It is observed that the reduction of material stiffness is generally due to the evolution of defects such as cavities, cracks, etc. These zones cannot support tensile stresses. It is proposed to characterize damaged material only with the property that the stress vanishes in the damaged zone. It is necessary to distinguish between two different zones : the sound elastoplastic material with volume Ω and the damaged one where the stresses are identically equal to zero. The previous results obtained in [23] is then recovered. In particular a relation between the global tangent modulus and the local one is obtained in the form:

(57) $$\dot{\Sigma }:\dot{E}=<\dot{\sigma }:\dot{\varepsilon }>-\int_{\Gamma }\nabla w.\underline{n}\phi^{2}dS$$

This condition gives us a condition of stability in this case as pointed out in [30].

## 6. Examples and Applications

### 6.1. Damage model and CSA

The composite spheres assemblage of Hashin is analyzed in [10]. In this paper, the rate boundary value problem, when a criterion of propagation of the interface is given in terms of an energy release rate, is discussed.

The system is composed by a compact assemblage of spheres with external radii in order to fill the whole domain. The microscopic structure is constituted by composite spheres with a core made with material 2 and the shell by material 1, both materials are homogeneous and linear elastic. As in the general case, material 1 transforms into material 2 ; the transformation is irreversible and the criterion is a generalized Griffith’s one based on the energy release rate of the transformation. The volume fraction of material 2 is denoted by *c*. Applying the same method than in [28], the assemblage is considered well-disordered. Using the particular three phase model of [29], the homogeneous equivalent medium denoted by material 0 is unknown. In phase *i* the local characteristics are the bulk modulus denoted by $k_{i}$ and shear modulus by $\mu_{i}$. In what follows $k_{1}$ is assumed to be larger than $k_{2}.$

A generalized Griffith law is given to govern the transformation

(58) $$G<G_{c},\phi =0\;;G=G_{c},\phi \geq 0$$

**Figure 1:**
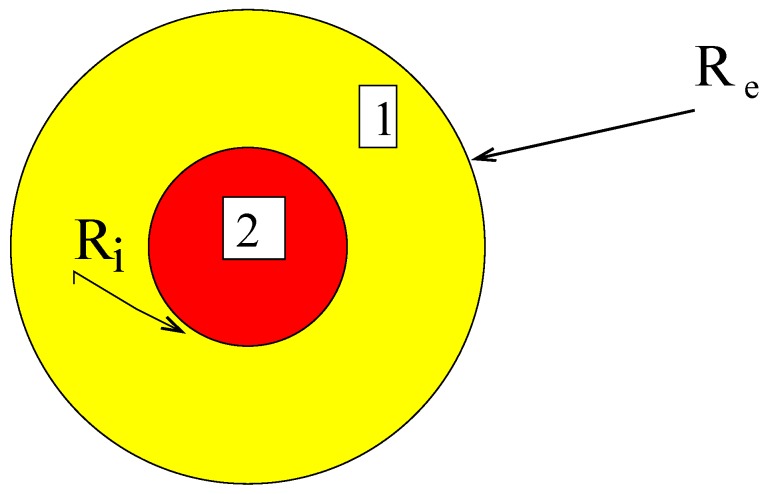
The composite sphere.

The composite sphere is submitted to an isotropic loading, the radial displacement is prescribed on the external boundary ($R=R_{e}$). For isotropic linear elasticity the solution is determined considering a radial displacement

(59) $$\underline{u}=u_{i}\left(R\right){\underline{e}}_{r},u_{i}\left(R\right)=A_{i}R+\frac{B_{i}}{R^{2}},\;i=1,2$$

The boundary conditions imply:

(60) $$u_{1}\left(R_{e}\right)=E\;R_{e},u_{2}\left(0\right)=0,B_{2}=0$$

For a given history of *E*, the external surface is submitted to a radial force:

(61) $$\sigma_{1}\left(R_{e}\right).{\underline{e}}_{r}=\Sigma {\underline{e}}_{r}$$

For given volume fraction of material 2, $c=\frac{R_{i}^{3}}{R_{e}^{3}}$, the solution of heterogeneous elastic sphere is

$$\begin{array}{ccc} b & = & \frac{B_{1}}{R_{i}^{3}}=\frac{3\left(\kappa_{1}-\kappa_{2}\right)A_{1}}{3\kappa_{2}+4\mu_{1}}\\ E & = & \frac{D(c)}{3\kappa_{2}+4\mu_{1}}\;A_{1}\\ \Sigma & = & \sigma_{rr}\left(R_{e}\right)=\frac{3E}{D(c)}\left(\left(3\kappa_{2}+4\mu_{1}\right)\kappa_{1}-4\mu_{1}c\left(\kappa_{1}-\kappa_{2}\right)\right) \end{array}$$

where

$$D\left(c\right)=3\kappa_{2}+4\mu_{1}+3c\left(\kappa_{1}-\kappa_{2}\right),\;c=\frac{R_{i}^{3}}{R_{e}^{3}}$$

The last equation defines the global behaviour of the composite sphere as having an effective bulk modulus

(62) $$\kappa^{eff}=\frac{\left(3\kappa_{2}+4\mu_{1}\right)\kappa_{1}-4\mu_{1}c\left(\kappa_{1}-\kappa_{2}\right)}{3\kappa_{2}+4\mu_{1}+3c\left(\kappa_{1}-\kappa_{2}\right)}$$

Then it is obvious that when *c* tends to zero, $\kappa^{eff}$ tends to $\kappa_{1}$.

When the radius $R_{i}$ increases, the rigidity of the composite sphere decreases and some dissipation occurs. The dissipation is given by the rate

(63) $$4\pi R_{i}^{2}G\left(R_{i},E\right){\dot{R}}_{i}=-\frac{\partial W}{\partial R_{i}}{\dot{R}}_{i}$$

This defines the energy release rate associated with the dissipation along a moving surface [24].

Along the interface Γ the energy release rate has the value

(64) $$G\left(R_{i},E\right)=\frac{9E^{2}}{D^{2}\left(c\right)}\left(\kappa_{1}-\kappa_{2}\right)\left(3\kappa_{2}+6\mu_{1}\right)\left(3\kappa_{1}+4\mu_{1}\right)$$

#### The response under monotonic loading

The loading parameter *E* is increasing. Initially, the core does not evolve, the critical value $G_{c}$ is not reached. At one time the critical value $G_{c}$ is reached, the strain is $E_{c}\left(c_{o}\right)$. After that the actual value of $R_{i}$ is determined by the implicit equation

(65) $$G\left(R_{i}\left(t\right),E\left(t\right)\right)=G_{c}$$

this is the consistency condition. During this phase the internal radius $R_{i}$ increases monotonically with *E* and attains the value $R_{e}$ at the value $E_{T}$ of the loading.

To any chosen critical value $G_{c}$ corresponds a Griffith type local criterion for fracture, and this induces that the local stretch $u\left(R_{i}\right)/R_{i}$ is a constant proportional of the square root of $G_{c}$. From equations 64 and 65 we deduce that when the damage occurs

(66) $$\frac{E}{D(c)}=\alpha_{c}$$

where $\alpha_{c}$ is a constant. We remark that *D* is an increasing function of *c*. During the damage evolution $A_{1}=E_{c}$

(67) $$\alpha_{c}=\frac{E_{c}}{3\kappa_{2}+4\mu_{1}}=\frac{1}{3\kappa_{1}+4\mu_{1}}\frac{u(R_{i})}{R_{i}}.$$

**Figure 2:**
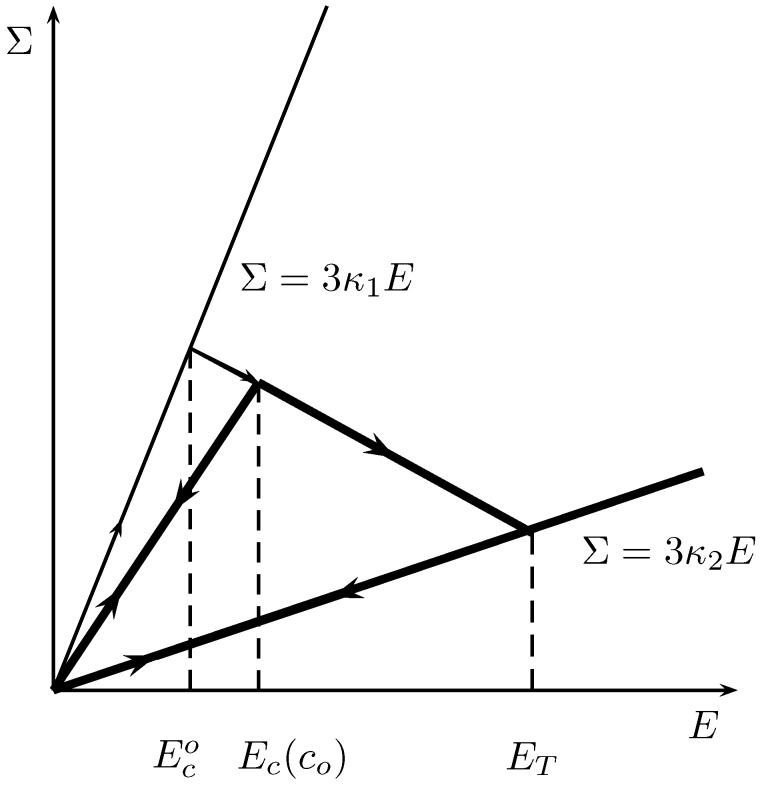
The response of the composite sphere with local damage.

At intermediate time t<T the sphere is not completely transformed, $G\left(R_{i}\left(t\right),E\right)<G_{c}$ for any E<E(t), then the composite sphere has the answer of an elastic heterogeneous medium with new concentration $c=R_{i}^{3}/R_{e}^{3}$. The global bulk modulus decreases with the transformation. With this propagation law of the interface, from it’s initial position determined by $c_{o}=R_{i}^{3}\left(O\right)/R_{e}^{3}$, we have successively for an increasing function E(t):

(68) $$\left\{\begin{array}{cc} E\left(t\right)<E_{c}\left(c_{o}\right),\;G\left(R_{i},E\left(t\right)\right)<G_{c}, & R_{i}\left(t\right)=R_{i}\left(0\right)\\ E\left(t\right)\geq E_{c}\left(c_{o}\right),\;G\left(R_{i}\left(t\right),E\left(t\right)\right)=G_{c}, & R_{i}\left(t\right)=f\left(E\left(t\right)\right)\\ E\left(t\right)=E_{T},\;G\left(R_{e},E_{T}\right)=G_{c} & R_{i}\left(T\right)=R_{e}\\ E\left(t\right)\geq E_{T}, & R_{i}\left(t\right)=R_{e} \end{array}\right.$$

and the answer can be plotted as in Fig.1 Now we consider the macroscopic behaviour of a composite spheres assemblage when two families exist in the structure, with volume fraction of phase 2 denoted by $c_{I}$ and $c_{II}$ $\left(c_{I}>c_{II}\right)$. For any given macroscopic *E*, we can show in an analytical way that

(69) $$\left(G_{I}-G_{II}\right)\left(c_{I}-c_{II}\right)\left(\mu_{1}-\mu_{2}\right)>0$$

As previously at the beginning of the loading the macroscopic behaviour is linear elastic, until the criterion of propagation is reached for one family. So we have the following cases:

- if $\mu_{1}>\mu_{2},$ the difference between the two concentrations $\left(c_{I}-c_{II}\right)$ increases until the larger reaches the value 1,
- if $\mu_{1}<\mu_{2}$, the difference between the two concentration decreases, than the assemblage tends to the assemblage of only one family.
- if $\mu_{1}=\mu_{2}$ both concentrations could increase.

So if we consider the assemblage of the two families as a perturbation of the assemblage of one family, this study can be considered as an analysis of bifurcation for small differences $(c_{I}-c_{II}).$ In the first case a new well disordered family can appear along the first one. So the answer of the global behaviour in such a case is not unique.

Even the system is composed by only one family of similar composite spheres, the local response to the loading increment is not unique. In fact, many kinds of bifurcations can exist. This shows the necessity to study stability and bifurcation of each equilibrium path in homogeneization of nonlinear mechanical behaviour to ensure the existence of the macroscopic law.

### 6.2. Variational procedures

The most recent procedures for predicting the overall nonlinear properties of composites are the variational procedures. The first contribution [31] were extended by many studies [32,33,34].

For nonlinear elasticity, in small strain, variational characterization are due to the convexity of the free energy relative to the strain.

Considered a composite made up of power-law materials with the same exponent *n* and the same reference strain $\varepsilon_{o}$ but different flow stress $\sigma_{o}$. The variational characterization of the effective behaviour is given by

(70) $$\Phi \left(E\right)=\underset{v}{\inf}\left(\frac{1}{1+m}\varepsilon_{o}^{m}<\sigma_{o}\left(x\right)\varepsilon_{eq}^{1+m}\left(v\left(x\right)\right)>\right)$$

where *v* satisfy boundary conditions v=E.x over ∂Ω, and $\varepsilon_{eq}=\sqrt{\frac{2}{3}\varepsilon :\varepsilon }$. Introducing a linear inhomogenous material, incompressible and isotropic with an arbitrary non-negative shear modulus μ(x) depending on the point position *x*

(71) $$\sigma_{o}\left(x\right)\varepsilon_{eq}^{1+m}={\left(\frac{3}{2}\mu \varepsilon_{eq}^{2}\right)}^{(m+1)/2}\sigma_{o}\left(x\right){\left(\frac{3}{2}\mu \right)}^{-(m+1)/2}$$

Using Hölder’s inequality on the function introduced in the last equation for the couple of function

$$f={\left(\frac{3}{2}\mu \varepsilon_{eq}^{2}\right)}^{(m+1)/2},\;g=\sigma_{o}\left(x\right){\left(\frac{3}{2}\mu \right)}^{-(m+1)/2}$$

and r=2/(m+1),s=2/(1-m), bounds are obtained ([33]) and optimum value is obtained using of the overall elastic energy of the comparison linear composite.

For isotropic incompressible two-phase material, it can be noticed that the prediction derived from the classical Hashin-Shtrickman upper bound is a rigorous bounds upper bounds for all isotropic composites and similarly, the prediction deduced form the HSZ upper bound is a rigorous upper bound for all isotropic CSA ([33]).

More general results and applications for nonlinear composite can be found in [36].

## 7. Conclusions

This paper shows how local mechanical behaviour can influence the global behavior of an heterogeneous medium. In all cases, the microstructure must be taken into account. In linear elasticity the macroscopic behaviour is defined by the spatial distribution of the phases, in nonlinear cases the evolution of the internal state contributes to the evolution of the microstructure. In the last case a complex boundary value problem must be solved. Even if the determination of a global quantity by average process guarantees the uniqueness of a macroscopic mechanical variable, the choice of the localization process is very important. The choice of this process must ensured the local response is unique when the external loading evolves. The study of the condition of stability and non-bifurcation is emphasized in order to be able to define the macroscopic behaviour, some cases of non-uniqueness have been given.

The role of the incompatible internal strains or stresses have been presented. In the case of partially damaged materials defined by a transformation along a moving surface, even if at local scale the components are linear elastic, the macroscopic behavior is no more non-dissipative. When initially there exists strains and self equilibrated stresses in the structure, the propagation of the interface will increase their influence on the mechanical macroscopic behavior.

Extension in finite strain can be given with application to polycrystals. The macroscopic law of a polycrystal have the same form of the micro one and we underlined that the decomposition in elastic and plastic parts of the behaviour is determined by the definition of the orientation of the polycrystal. This orientation is given as an average quantities of the orientation of the local crystals. Taking the definition of this orientation into account represents some information of the microstructure.[37]
